# Evaluating the impact of a training program to support transitioning from the hospital to the community for people after stroke: a community case study

**DOI:** 10.1186/s12913-021-07436-7

**Published:** 2022-01-05

**Authors:** Michelle Lui, Katherine McKellar, Shari Cooper, Janice J. Eng, Marie-Louise Bird

**Affiliations:** 1 Department of Occupational Science and Occupational Therapy, Faculty of Medicine, University of British Columbia, 2211 Wesbrook Mall T325, Vancouver, BC V6T 2B5 Canada; 2 Kelowna General Hospital, 2268 Pandosy Street, Kelowna, BC V1Y 1T2 Canada; 3 Parkinson’s Recreation Centre, 1800 Parkinsons way, Kelowna, V17 4P9 Canada; 4 Department of Physical Therapy, Faculty of Medicine, University of British Columbia, 212-2177 Wesbrook Mall, Vancouver, BC V6T 1Z3 Canada; 5 Rehabilitation Research Program, GF Strong Rehabilitation Research Laboratory, Vancouver Coastal Health Research Institute, 4255 Laurel Street, Vancouver, BC V5Z 2G9 Canada; 6 School of Health Sciences, University of Tasmania, Newnham Drive, Launceston, Tasmania 7250 Australia

**Keywords:** Stroke rehabilitation, Transitions, Community navigation, Physiotherapy, Exercise, Implementation science

## Abstract

**Background:**

The transitions in care along the stroke recovery path are challenging, particularly in finding mechanisms to continue one’s recovery once at home. We aim to evaluate the impact of training physiotherapists and fitness instructors from one regional community together to deliver an evidence-based group exercise program starting in the hospital and transitioning to the community using an implementation approach.

**Methods:**

The evidenced based exercise program Fitness and Mobility Exercise (FAME) for stroke was chosen as the intervention. Data from interviews with stakeholders (community centre and health authority hospital staff including a physiotherapy navigator) was transcribed and themes evaluated using the RE-AIM (Reach, Efficacy, Adoption, Implementation, Maintenance) framework. These data were supplemented by information collected as a quality assurance project within the health authority.

**Results:**

Two programs were established; one in the community centre (run over 15 months by fitness instructors) and one in the regional hospital (run over 12 months by a rehabilitation assistant under the direction from a physiotherapist). Transitions in care were facilitated by implementing the same evidence-based group exercise class in both the hospital and community setting, so people living with stroke could seamlessly move from one to another. An existing physiotherapist navigator service also was valued as a support for the transitions between the two centres for people with stroke. The hospital group accessed group-based physiotherapy service on average 31 days earlier than they were able to in a one-to-one format.

**Conclusions:**

This case study described the implementation of the Fitness and Mobility Exercise (FAME) program in one community and the use of a physiotherapist navigator to assist transition between them. After a community training workshop, FAME programs were established within the health authority and the community centre. FAME program participants within the health authority benefited from reduced wait times to access hospital outpatient physiotherapy service. Improvements in function were measured in and reported by the people after stroke attending either the health authority or community centre FAME groups.

**Supplementary Information:**

The online version contains supplementary material available at 10.1186/s12913-021-07436-7.

## Background

Stroke rehabilitation health professionals and people living with stroke have identified that transitions in care along the stroke recovery path are a major gap that needs to be rectified [1]. The transition from hospital to home requires coordination from multiple centres or units to support individuals as they continue their physical, cognitive, and emotional recovery at home. As one mechanism of continuing their recovery, people with stroke are encouraged to participate in evidence-based community exercise programs [2]. The importance of physical activity for reducing secondary risk factors for another stroke has been well established [3]. It is known that physical function and mobility are positively associated with physical activity [4] with evidence to show that community-based group-exercise interventions improve and retain mobility, functional capacity, and balance [5]. Furthermore, community-based group-exercise interventions motivate individuals to leave their homes; to connect socially; to establish structure and routine; to advance personal growth and development while also supporting and influencing others to accomplish similar goals [6]. As such, supporting stroke survivors to participate in physical activity is an identified international priority [7]. However, sedentary time is prevalent following stroke [8] and is of great concern as it limits functional improvement and increases cardiovascular risk [9].

There are multiple barriers to participation for people after stroke that relate to individual and systems level issues. From an individual perspective, motivation and confidence, physical disability, support, and transport are important. Lack of availability and access to programs and models to support transitions in care on a systems level pose barriers to participation [10–12]. In a recent knowledge translation study with healthcare, community and consumer stakeholders, navigation to community services was described as an unmet need [13]. While a partnership between the health service sector and the community centers can facilitate referral pathways to support individuals transitioning out of the health system to be reintegrated into the community, examples of this are rare.

The purpose of this study is to evaluate the impact of training physiotherapists and fitness instructors from one community to deliver an exercise program for people after stroke starting in the hospital and transitioning to the community based on the Fitness and Mobility Exercise (FAME) program using the RE-AIM (Reach, Efficacy, Adoption, Implementation, Maintenance) framework [14]. These elements have been interpreted in line with recent descriptions by the original authors [15] (see Table 1 for more detail). RE-AIM has been widely used in planning and evaluation of health program and policies [16]. The system impact was evaluated upstream (re-organization of hospital services), as well as downstream (e.g., coordination of community centre referral pathway). This evaluation can inform considerations for what, where, when and how to deliver effective, evidence-based rehabilitation interventions and community programs for this population and as a model for programs for other chronic health conditions.

**Table 1:** RE-AIM framework data collection methods and interpretation

RE-AIM ELEMENTS	Interpretation	Data Tool Used
Reach	Number, proportion of individuals willing to participate, use of strategies to improve access and awareness	Hospital audit data Qualitative interviews
Effectiveness	Impact of intervention on important outcomes, including quality of life and unintended outcomes	Hospital quality assurance data. Exit survey data, Community centre screening.
Adoption	Number of people and settings who are willing to deliver the program	Qualitative interviews
Implementation	Program fidelity and any adaptations made, consistency and time required.	Qualitative interviews
Maintenance	Extent to which the program is sustained at more than 6 months after implementation, any changes to policy or systems, reasons for discontinuation	Qualitative interviews

## Methods

### Intervention

Potential delivery of a FAME program training workshop was discussed by telephone with a regional hospital manager, community centre programmer, and physiotherapist navigator, who were all supportive. A full-day workshop was delivered in September 2017 to teach the FAME Program to physiotherapists (who could implement the program in the hospital stroke physiotherapy program) and fitness instructors (who could implement the program in the community centre facility). Three participants with stroke acted as demonstrators for hands-on practice during the workshop. The workshop was delivered in the September 2017.

### Participants

Study participants were invited either from a list who had previously attended training (physiotherapists, fitness instructors) to deliver community-based exercise for people after stroke (FAME), or identified from participants as being key stakeholders (e.g., managers with the community centre and health authority or having experience in delivering the FAME program). No study participants who were invited, declined to be involved. Ethical approval for the study was granted by the University of British Columbia and the Interior Health Quality Improvement and Patient Safety Office and FAME program participants provided written consent.

### Data collection

A series of three stakeholder interviews were held approximately 21 months after the original workshop (July 2019). The first was held with the physiotherapist navigator, the second with staff employed at the community center (program manager and instructor delivering FAME), and the third with health authority staff (Physiotherapy Professional Practice Leader and Physiotherapy Manager).

Semi-structured qualitative interviews were held with staff at the health authority and community centre in a face-to-face format using a pre-determined interview schedule focused on implementation factors (e.g., drivers for change, fidelity and adaptation, barriers and facilitators). The researcher conducting the interviews was a rehabilitation clinician-scientist with a background in physiotherapy. All interviews were digitally recorded with the participant’s consent, with field notes taken during the interviews to add richness to the data and provide context.

Data were supplemented by evaluation and participant feedback gathered from the hospital as part of a quality improvement evaluation during the third phase of the implementation (December 2019 to March 2020) and by a site visit by one of the authors (MLB) to a class at the community centre in July 2019. Outcome data included from the health authority includes pre- and post-FAME measures for ambulation status, 10 m Walk Test, Timed Up and Go (TUG) [17], and Berg Balance Scale (BBS) [18]. Patients on the outpatient neurological rehabilitation program waitlist were screened between December 2019 and March 2020. Outcome data were collected on all enrolled patients by senior physiotherapy staff prior to commencement and then on discharge from the FAME program. Any missing data were treated with imputation. An exit survey was delivered by the senior physiotherapist who was not running the classes. The survey included a number of closed and open-ended questions (see supplemental file). The closed questions used a 5-point Likert scale (strongly agree to strongly disagree) to report agreement with a set of statements. Data were analyzed and number of positive, neutral, or negative responses were counted. After each question participants had the opportunity to provide comments. Anonymously, quotes from the open-ended survey questions were selected to illustrate the domains of the REAIM framework.

The Short Physical Performance Battery was used to collect changes in function at the community centre [19]. These data were collected as part of pre-screening at entry to the program and designed to be used to give feedback to program participants on any changes in physical functioning that occurred during their participation.

### Data analysis

All interviews were recorded, transcribed, and analyzed using standardized qualitative methods. Two analysts (ML and MLB) coded all of the data independently. Data were read and re-read and codes developed and agreed on by the research team. The RE-AIM framework [14] was used to inductively interpret the themes that emerged, and then discussed them with all team members. Field notes from the interviews were used to triangulate the data.

This framework includes elements of Reach, Efficacy, Adoption, Implementation and Maintenance. Interpretation on these elements and the use of the different data collection tools is outlined in Table 1.

The first analyst is a female occupational therapist with experience working with a neurological population in the acute and post-acute phase. The second is a female clinician scientist with a background in physiotherapy and more than 10 years of experience in qualitative research. The team met to discuss and reach consensus on the meanings or themes that relate the implementation of the exercise program. Quotes from stroke participants are labeled with a P (e.g., P1, P2…); health authority physiotherapists with the abbreviation PT, and community centre staff labeled with CC. Changes in physical functioning (pre-post mean, standard deviation) were analyzed using Student T-test in excel.

### Exercise program

FAME is a cost-effective, evidence-based group exercise program that can be implemented into community or hospital settings for people post-stroke. The hour-long classes include exercises that aim to improve walking and mobility through a focus on functional strength, balance and fitness. Clinical trials have found high levels of effectiveness with FAME to improve motor function (muscle strength, balance, walking), cardiovascular fitness, and bone density in the stroke population [20–22]. The FAME program components can be downloaded from www.fameexercise.com.

## Results

The workshop was attended by three health authority physiotherapists and twelve community centre fitness instructors. Of these, one physiotherapist and one fitness instructor went on to teach the FAME program to people with stroke, with another physiotherapist assisting with screening and evaluation. Fitness instructors were eager to take the course despite knowing that the community centre would have limited instructors for the course. The skills gained in the workshop were applicable to other courses they taught, as well as for personal training with clients.

Following the workshop training, FAME classes were delivered twice a week for one-hour each in both the hospital and the community centre. Group classes at the hospital were provided at no cost to people while they were on the waitlist to receive one-to-one outpatient physiotherapy. As spots became available in the FAME class in the health authority, new participants were added. Hospital classes were delivered in a maximum of one instructor to two participants ratio.

Classes at the community centre were run in line with the schedule at the centre (around school terms) at a cost-recovery basis of $10 Canadian. The community centre has options for funding relief for those clients who are not able to afford the classes. Community centre classes were delivered in a maximum of one instructor to four participants ratio.

We have applied the RE-AIM framework to describe the implementation of an exercise program for people after stroke in the community that is supported by:

1. Training of the centre fitness instructors and the health authority physiotherapists together.
2. Implementation of FAME classes within the health authority as well as the community centre.
3. Assistance of a physiotherapist who works as a community navigator out of the community centre for 90 minutes, three times a week, to help transition clients from hospital services to community-based exercise.
4. Assistance of local university students who supported the running of the program at both the hospital and community centre site by providing exercise encouragement, supervision, and stand-by assistance, and recording attendance. In the hospital, students helped to teach the exercises to the participants and helped set up the physical space for the class.

### Reach (hospital)

**Fig. 1:**
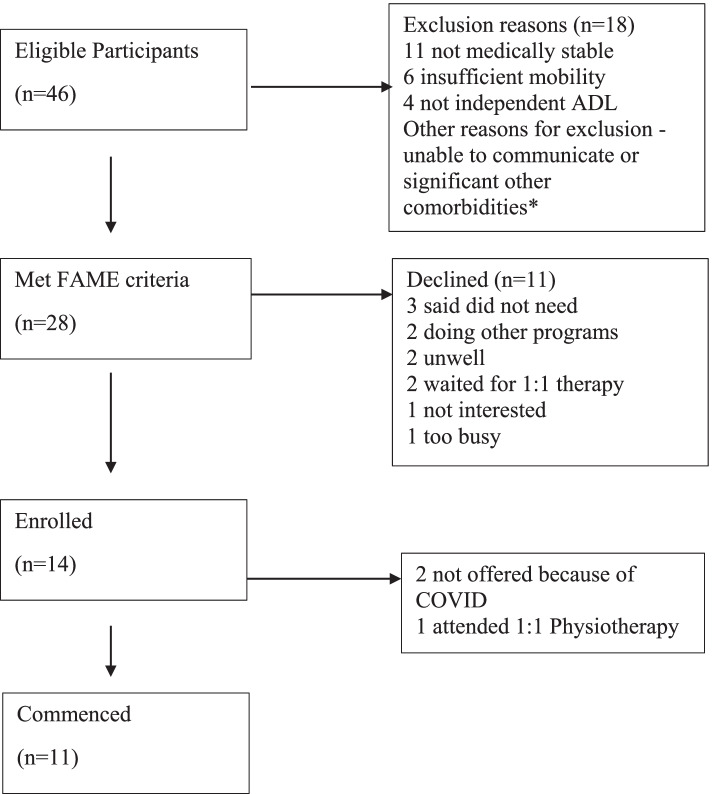
Flow of participants through the hospital outpatient neurological rehabilitation program. *several people had multiple reasons for being excluded

This health facility admits over 400 people with stroke annually [23]. During the time frame of data collection for this study there were 46 possible candidates – patients with a diagnosis of stroke or acquired brain injury referred to outpatient neurological physiotherapy who met the criteria for the FAME exercise program (i.e., medically stable; able to stand 5 min; walk 10 m with no more than standby assistance; communicate and follow directions; and toilet independently). Enrollment flow is described in Fig. 1.

Half of the eligible participants attended an assessment session (14/28). Eleven participants participated in FAME classes, with a mean age of 69.5 years and a median age of 72 years (ranging between 31 to 87 years old). There were five males (45%), six females (55%). Ninety-one percent had a diagnosis of stroke, 9 % were neurosurgical, none had other types of ABI. Participants were an average of 44.5 days post-event (ranging between 23 to 77 days) before they started participating in the FAME program.

### Reach (community Centre)

The community centre used various media to reach participants. Printed and web-based program guides advertised relevant community centre programs. Targeted guides for seniors were distributed at the local seniors centre.

Prior to this study, the health authority had been collaborating with the local community centre via a physiotherapist navigator who provided service 3 days a week for 90 minutes at a time to screen and advise clients on appropriate programs. With the new addition of a stroke specific class at the community centre, the physiotherapist navigator could now provide an option for people after stroke to transition to those classes at the centre. Access to the program was improved by the use of student volunteers to provided additional supervision for those who needed it;

“So we will put in the request again ...as this is the program that could use constant volunteers. And then we will be able to maybe allow a few more [program participants] in, only if those volunteers are in place’ [CC1].

As this physiotherapy navigator also worked at the hospital outpatient program, the element of familiarity (‘familiar face’ of the clinician along with the clinician’s familiarity of the participants’ function) was described to be beneficial in establishing a plan for continuity of therapy post-discharge. Sixteen participants attended the program. No participants were excluded. One other potential participant visited but did not enroll. Having a physiotherapy navigator was noted to be an effective service;

‘…we have really good buy in by the navigators, the physiotherapists who come here 3 or 4 times a week and they use this as their office, and they meet with people and refer them’ [CC2].

Facilities tried to mitigate cost-based barriers to access the programs.

‘Financially we try to do away with the barrier for people with the access pass or we have recreational opportunity coupons that we can give people if they are financially disadvantaged. We just want to take away all the barriers that would keep them from coming’ [CC2].

### Efficacy (hospital)

Although they waited an average of 57 days for individual PT intervention, eligible clients were able to enter the FAME program an average of 26 days after hospital discharge. This meant that they could participate in the FAME-based group PT intervention rather than no intervention at all during the wait time for individual physiotherapy.

There were improvements in balance and walking, with significance reached for balance function (*n* = 11; see Table 2). In addition to the measured clinical benefits, participants provided positive feedback on the program. Six participants (two males and four females, response rate of 55.4%) completed an anonymous questionnaire about the FAME group. All six participants agreed or strongly agreed with the following statements:

- The exercise class met my expectations.
- I felt safe in the class.
- I was able to access the facility easily.
- I progressed in the number of repetitions of the exercises over the course of the program.
- I progressed in the difficulty level of the exercises over the course of the program.
- The exercises were intense enough to improve my function.
- The instructors were encouraging in the class.
- The group met my needs while I was waiting for a spot in the full outpatient neuro rehab program.

**Table 2:** The efficacy of the program for people after stroke in the health authority (hospital) and community centre

	Health Authority (Hospital)	Community Centre
Efficacy^a^	Data from third iteration (baseline *n* = 11) (mean change and 95%CI) TUG −2.6 s (− 6.7 to 1.5) *p* = 0.2 10 Meter walk (− 1.6 s) -4.3 to 1.1, *p* = 0.23 Berg Balance Scale 4.3 (0.9 to 8.5) *p* = 0.05 One participant stopped using mobility devices. Three did not require physiotherapy at end of FAME. Clients accessed physiotherapy service, albeit in a group format, an average of 31 days earlier than they were able to get in for 1:1 PT.	Changes in Short Physical Performance Battery (*n* = 16) 1.6 (− 0.8 to 4.0) *p* = 0.17 Two participants stopped using mobility devices.

^a^There were four missing data points treated with imputation by last value carried forward

Five of the six participants agreed or strongly agreed that ‘The group helped smooth the transition from hospital to home. Two participants (33%) responded neutrally to the question that ‘The group helped me cope with my stroke or brain injury.’

Comments on the questionnaire mostly related to the atmosphere of the class and the physical space. Participants enjoyed the instructors and felt well supervised. For example, ‘Very positive instructors’ [P1], and ‘I felt safe at all times’ [P2]. The camaraderie of the class was very positive as well, as evidenced by the following quotes, ‘Friendship and great work out’ [P3] and ‘Being with others in the same situation’ [P2]. Safety considerations were recognized by participants with the comment, ‘Usually always a spotter on the tougher stations’ [P3]. There were no adverse events reported.

### Efficacy (community Centre)

Improvements in physical function were recorded by the Short Physical Performance Battery (see Table 1). While not statistically significant, improvements in mobility were described as important to the participants.

‘Freya(not real name) used to be in my Get Up & Move, but it really wasn’t giving her what she needed so actually my other people that are in the FAME program talked to her into coming.’ They said ‘You need to come to this cause you're going to get more benefit in the FAME program than what we are getting in the Get Up & Go program. So she did and it's made a huge difference. Huge difference in her progress’ [CC1].

### Adoption (hospital)

Of the three hospital physiotherapists who attended the training, one had the dual role of being a physiotherapy navigator and a therapist in the outpatient neurological rehabilitation program. Another works in the outpatient neurological rehabilitation program who planned and coordinated the evaluations described here. The third was not involved in FAME after the training, due to staff rotation within the health service. There were adaptations over a one-year period. The program started with the physiotherapist and rehabilitation assistant facilitating classes for existing outpatient clients. By the third iteration, a physiotherapist screened clients on the outpatient neurological physiotherapy waitlist, and those who met eligibility criteria and consented to group intervention enrolled in the FAME classes run by a rehabilitation assistant and two kinesiology student volunteers.

### Adoption (community Centre)

Of the twelve fitness instructors who were trained initially, one was selected to run the FAME program in the community centre in the city. The community centre manager described the transient and part-time nature of the staff who worked there as a challenge to the long-term sustainability of specialized classes. In view of providing FAME classes at a second community centre also managed through this venue, the manager was supportive of having the existing fitness instructor use a workplace-training model to train other fitness instructors at the centre with a view to expanding to the second site in the future.

### Implementation (hospital)

Participants attended a mean of 6.9 FAME classes (ranging between two to ten classes). Each class had three to six participants, with a mean of 4.7 participants (with multiple instructors as required to maintain a 1 instructor to 2 participant ratio). Attendance was excellent, with only four absences in sixteen classes.

Initially, screening for the FAME program was undertaken by the outpatient physiotherapist, and the program was run by a rehabilitation assistant with assistance from student volunteers. The hospital developed an abbreviated FAME education program to upskill university students to the FAME program in order to assist with running the program. Classes were capped at six participants to ensure appropriate supervision and adequate physical space. Furniture was moved to facilitate an open area with free wall space while a railing was installed along one wall for support during exercises. Clinical observation and vital signs monitoring were added as many of these sub-acute FAME program participants have cardiac or respiratory concerns. One study participant identified added benefits of providing the FAME program participants with education on how to self monitor,in terms of transition to the community:

‘I think one of those transition things that as they are leaving, and having to become independent in the community, you need them to be able to self-monitor [PT1].

The rehabilitation assistant developed a set of group planning sheets so that the instructor could rotate through these pre-determined programs with a different set of exercises each time. Using dry-erase markers, the participants initialed the laminated exercise sheets at each station to ensure that they completed all stations. Partway through the program, inpatient rehabilitation physiotherapists participated in some in-house training and subsequently took over the role of pre-screening for FAME, which saved time for the outpatient therapist to facilitate earlier entry into the program.

### Implementation (community Centre)

The program was implemented in line with the training provided. However, given several participants had reduced mobility, adaptations were made to meet their needs. Initially, the programs were delivered with all participants doing the same exercises until the instructor felt that they were competent and could perform the exercises safely and in good form. The instructor then set up a circuit style class that the participants rotated through, using printed handouts from the manual supplied in training to identify which exercises were to be performed at each station. That instructor was able to individualize the exercise then;

‘Well I just had to add extra stuff in for people over and above the level of the exercises so had to add in harder stuff for some of the participants” [CC1].

Although several clients had significant aphasia, the instructor was able to include these individuals with support from a university kinesiology student.

### Maintenance (hospital)

FAME classes ran from April 2019 to March 2020 in three phases. After March 2020, in light of the COVID-19 pandemic, FAME was modified to be delivered by a rehabilitation assistant in a physically distanced format with two participants. Classes did not run regularly.

### Maintenance (community Centre)

FAME classes ran from February 2018 to March 2020 (with a break for the summer). Three participants transitioned to non-stroke classes. Since then, FAME classes have been on hold in response to the COVID-19 pandemic public health orders including restrictions on indoor gatherings and group exercise classes.

## Discussion

This paper reported on the implementation of FAME, an evidence-based group exercise program for people after stroke within a hospital and a community centre environment. Through the provision of a training workshop to health professionals and fitness instructors, we have described how one community has overcome a range of system-wide and individual barriers in supporting physical activity for people after stroke during the challenging transition from being a hospital patient to a community member.

Stroke rehabilitation is becoming more complex, with a greater number of transitions throughout the care continuum [24]. It is known that transitioning from hospital to home after stroke can present multiple changes and emotional and physical challenges to patients, families, and their caregivers, leaving individuals feeling overwhelmed and unprepared to reintegrate into their home and community [2]. A recent Canadian survey reported that only one-third of physiotherapists consistently provided information on community exercise programs to patients with stroke [25]. The time of discharge may not always be the right time for this information about ongoing recovery, as patients and caregivers describe being overloaded with information then [24].

To overcome barriers to ongoing care, community navigation models have been used in the context of multiple health conditions [26, 27], including stroke management [28]. Navigators can be professionals or lay people who provide education; assist with financial barriers; aid in care coordination; make referrals to community resources; and provide emotional support [27]. A necessary component in making FAME implementation successful was the use of a physiotherapist navigator service, which has been highlighted in this community case-study to a be a positive example of bridging the transition from outpatient rehabilitation and into the community with a physiotherapist a navigator to support this process. This is in line with a 2014 systematic review which has found that individualized tailored counselling motivated and helped people overcome barriers to participation, leading to improvements in physical activity [29]. Consistent with themes identified in the literature whereby professional navigators were valued for their clinical expertise, knowledge of the system, and understanding of the patient needs [30], the role of a physiotherapist navigator was described to be effective in our case-study. Specifically, the therapist’s knowledge of the programs, services and being a link between health service and community centres, facilitated connections of individuals to community resources.

Implementation of FAME in this community has resulted in positive upstream and downstream effect; namely reducing outpatient load and enhancing efficiency and access to outpatient rehabilitation post-discharge, thus noted a trend to reduce the need for individual physiotherapy after participation in FAME. From a process perspective, the outpatient therapy program delivering FAME found it beneficial for the inpatient unit to screen the participants for FAME since they would be most familiar with these individuals. The subsequent enhanced coordination in the hospital demonstrated the benefits of organization and communication between units. Through FAME training, knowledge and understanding of stroke was enhanced particularly for community centre staff, allowing the community centre to run a program for clients with specialized needs, in line with findings in the literature that stroke survivors prefer facility-based group exercise [31].

Beyond smoothing the transition between hospital and home and addressing their needs while awaiting outpatient rehabilitation, FAME participants expressed increased confidence in exercising through practice, supervision, and coaching. Beliefs about physical activity and self-efficacy are known to be important factors in facilitating physical activity after stroke [4] and predicting ongoing participation [32]. Notably, there was a high uptake and adherence to the FAME program with participants reporting feelings of positivity, camaraderie and belonging. As noted in the literature, components such as social support within programs, structured exercise, individualized instruction and reinforcing successful performance of exercise, have all been shown to improve uptake of exercise for people after stroke [10, 33].

Though FAME implementation was successful, there were some inefficiencies. Of note, only one of twelve fitness instructors went on to formally teach the program. It would have been ideal to rotate instructors to enable a small number to gain the skills in teaching the class to facilitate sustainability of the program. Rotation of physiotherapy and rehabilitation assistant staff through outpatient services also required ongoing training and the workload associated with this needs to be considered as part of implementation in other communities looking to use this as a model of service redesign. The advent of COVID-19 has changed the nature and feasibility of group exercise; hence, we were unable to measure ongoing effectiveness and sustainability of this intervention. Apart from training more instructors, utilizing university student volunteers has the potential to further increase efficiency and may even encourage some of these students to work in this area in the future – a strategy to recruit prospective clinicians across the province.

### Limitations

This is a small uncontrolled study, with variation in the timeframe for collecting physical outcome data (i.e., time spent on the rehabilitation ward and in the community programs varied) impacting how the improvements in physical outcome measures should be interpreted. As well, the described changes over time may include natural improvements. While our timeframe allowed us to collect program data at a considerable time period after the initial training (21 months) we did not collect long term data on FAME program participants, which impedes our ability to describe maintenance at an individual level. The risk of sampling bias from the small number of people with stroke who completed the exit survey is high.

## Conclusion

This community case-study showcased the important role of a physiotherapist navigator in effecting positive outcomes on the implementation of the FAME program. Training health and community centre staff together promoted safe implementation of FAME in both contexts simultaneously and addressed some systemic issues that were barriers to community participation. We established a link between clinical and community centre staff along with establishing continuity and familiarity for patients transitioning from the hospital to community programs. Participants benefited from reduced wait times to access hospital outpatient physiotherapy service. Further, this paper highlighted the impact that implementing evidenced based programs can have on extending traditional rehabilitation services, while having a physiotherapist navigator can foster an environment supportive of active engagement across transitions and continued recovery post-stroke.

## Supplementary Information

**Additional file 1.**

## Data Availability

The datasets used and/or analysed during the current study available from the corresponding author on reasonable request.
