# Supplementary material for: Evaluating the impact of a training program to support transitioning from the hospital to the community for people after stroke: a community case study
Source: BMC Health Serv Res. 2022 Jan 5;22:30. doi: 10.1186/s12913-021-07436-7 (PMC8729091; doi:10.1186/s12913-021-07436-7)
Supplement: Supplementary file 1 — Additional file 1. [file 12913_2021_7436_MOESM1_ESM.docx]

# **FAME Participant exit survey**

AGE (years): <20 20-29 30-39 40-49 50-59 60-69 70-79 80-89 90-99

Gender (circle one): M / F

Please rate how strongly you agree or disagree about the following statements by marking an ‘X’ in the correct box.

**1. The exercise class met my expectations.**

| Strongly Disagree | Disagree | Neutral | Agree | Strongly Agree |
| --- | --- | --- | --- | --- |

Comments:__________________________________________________________________________________________________________________________________________________________

**2. I felt safe in the class.**

| Strongly Disagree | Disagree | Neutral | Agree | Strongly Agree |
| --- | --- | --- | --- | --- |

Comments:__________________________________________________________________________________________________________________________________________________________

**3. I was able to access the facility easily.**

| Strongly Disagree | Disagree | Neutral | Agree | Strongly Agree |
| --- | --- | --- | --- | --- |

Comments:__________________________________________________________________________________________________________________________________________________________

**4. The exercises were intense enough to improve my function.**

| Strongly Disagree | Disagree | Neutral | Agree | Strongly Agree |
| --- | --- | --- | --- | --- |

Comments:__________________________________________________________________________________________________________________________________________________________

**5. I progressed in the number of repetitions of the exercises over the course of the program.**

| Strongly Disagree | Disagree | Neutral | Agree | Strongly Agree |
| --- | --- | --- | --- | --- |

Comments:__________________________________________________________________________________________________________________________________________________________

**6. I progressed in the difficulty level of the exercises over the course of the program.**

| Strongly Disagree | Disagree | Neutral | Agree | Strongly Agree |
| --- | --- | --- | --- | --- |

Comments:__________________________________________________________________________________________________________________________________________________________

**7. The instructors were encouraging in the class.**

| Strongly Disagree | Disagree | Neutral | Agree | Strongly Agree |
| --- | --- | --- | --- | --- |

Comments:__________________________________________________________________________________________________________________________________________________________

**8. The instructors listened to me and communicated well with me.**

| Strongly Disagree | Disagree | Neutral | Agree | Strongly Agree |
| --- | --- | --- | --- | --- |

Comments:__________________________________________________________________________________________________________________________________________________________

**9. The instructors included tips to help me live with stroke or brain injury.**

| Strongly Disagree | Disagree | Neutral | Agree | Strongly Agree |
| --- | --- | --- | --- | --- |

Comments:__________________________________________________________________________________________________________________________________________________________

**10. The group met my needs while I was waiting for a spot in the full outpatient neuro rehab program.**

| Strongly Disagree | Disagree | Neutral | Agree | Strongly Agree |
| --- | --- | --- | --- | --- |

Comments:__________________________________________________________________________________________________________________________________________________________

**11. The group helped smooth the transition from hospital to home.**

| Strongly Disagree | Disagree | Neutral | Agree | Strongly Agree |
| --- | --- | --- | --- | --- |

Comments:__________________________________________________________________________________________________________________________________________________________

**12. The group helped me cope with my stroke or brain injury.**

| Strongly Disagree | Disagree | Neutral | Agree | Strongly Agree |
| --- | --- | --- | --- | --- |

Comments:__________________________________________________________________________________________________________________________________________________________

**How could the class be improved?**

Comments:__________________________________________________________________________________________________________________________________________________________

**What was the best part of the class?**

Comments:__________________________________________________________________________________________________________________________________________________________

**Circle any that apply - Did you feel that you improved in your**

Fitness Balance Strength Walking Movement control

Comments:____________________________________________________________________________________________________________________________________________________________________________________________________________________________________________

**I chose to come to this group because**

The group was just for people with strokes and brain injuries

I wanted to improve my mobility

My physiotherapist recommended the group

Other _____________________________________________

**I decided to keep coming because**

I liked the group

I liked the exercises

I felt I improved

The instructors were well trained

Other _____________________________________________

Any last comments about the group:

______________________________________________________________________________________________________________________________________________________________________________________________________________________________________________________

*Thank you for your feedback!*
